# Radical Transfer Dissociation for De Novo Characterization of Modified Ribonucleic Acids by Mass Spectrometry

**DOI:** 10.1002/anie.201914275

**Published:** 2020-01-31

**Authors:** Giovanni Calderisi, Heidelinde Glasner, Kathrin Breuker

**Affiliations:** ^1^ Institut für Organische Chemie and Center for Molecular Biosciences Innsbruck (CMBI) Universität Innsbruck Innrain 80/82 6020 Innsbruck Austria

**Keywords:** dissociation, mass spectrometry, posttranscriptional modifications, radical chemistry, RNA

## Abstract

Mass spectrometry (MS) can reliably detect and localize all mass‐altering modifications of ribonucleic acids (RNA), but current MS approaches that allow for simultaneous de novo sequencing and modification analysis generally require specialized instrumentation. Here we report a novel RNA dissociation technique, radical transfer dissociation (RTD), that can be used for the comprehensive de novo characterization of ribonucleic acids and their posttranscriptional or synthetic modifications. We demonstrate full sequence coverage for RNA consisting of up to 39 nucleotides and show that RTD is especially useful for RNA with highly labile modifications such as 5‐hydroxymethylcytidine and 5‐formylcytidine.

## Graphical abstract

**Ready to dissociate**: Cobalt(III) hexammine can transfer hydrogen atoms to RNA anions and generate radical ions that dissociate into specific fragments for de novo characterization of modified RNA by mass spectrometry. This approach is especially useful for RNA with highly labile modifications, such as 5‐hydroxymethylcytidine and 5‐formylcytidine.

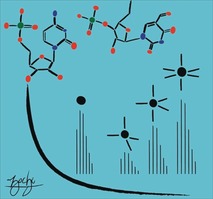

Posttranscriptional modifications of ribonucleic acids (RNA) play key roles in biological processes, but determining the function and significance of these chemically diverse (ca. 150) modifications with high‐throughput sequencing techniques (RNA‐Seq) alone can be quite challenging.1a Mass spectrometry (MS) of RNA is an emerging alternative approach as it can directly detect all mass‐altering modifications without the need for laborious sample preparation procedures.^1^ MS can be used at the nucleoside or nucleotide level for the identification and quantification—and at the oligonucleotide level for the identification, localization, and quantification—of posttranscriptional or synthetic modifications.1d, 2 In the “bottom‐up” approach, RNA is enzymatically digested into oligonucleotides for MS and MS/MS.2a Furthermore, “top‐down” MS of intact, undigested transfer RNA (tRNA, ca. 80 nt) has been demonstrated.^3^ Both top‐down and bottom‐up MS approaches utilize collisionally activated dissociation (CAD)^4^ of RNA into complementary ***c*** and ***y*** fragments formed by phosphodiester backbone bond cleavage (Scheme 1). Electron detachment dissociation (EDD) of RNA instead produces noncomplementary ***d*** and ***w*** fragments that differ in mass from ***c*** and ***y*** fragments by 18.011 and 79.966 Da, respectively.^5^ Since ***c*** and ***d*** fragments include the 5′ terminus, and ***y*** and ***w*** fragments the 3′ terminus, the analysis of only two spectra, one from CAD and one from EDD MS/MS, allows for de novo sequencing of completely unknown RNA with unknown modifications.3a, 6

**Scheme 1:**
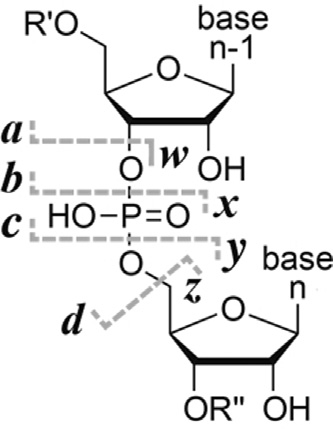
Nomenclature for fragments from RNA backbone cleavage (dashed lines indicate possible cleavage sites without implying a specific mechanism).

EDD of RNA,1d, 3a, 5 however, requires the use of Fourier transform ion cyclotron resonance (FT‐ICR) instruments in which (M−*n* H)^*n*−^ ions from electrospray ionization (ESI) can be irradiated with an electron beam (>20 eV) for production of (M−*n* H)^(*n*−1)−.^ radical ions by electron detachment.^7^ Alternatively, (M−*n* H)^(*n*−1)−.^ ions can be produced by electron photodetachment dissociation (EPD)^8^ using an ultraviolet laser, or by negative electron transfer dissociation (NETD)^9^ using reagent cations from a chemical ionization source. Here we report a new dissociation technique, radical transfer dissociation (RTD), that produces ***c***, ***d***, ***y***, and ***w*** fragments for de novo characterization of RNA in a single spectrum. In RTD, cobalt(III)hexamine ([Co^III^(NH_3_)_6_]^3+^) serves as the reagent for the production of RNA radical ions that dissociate into ***d*** and ***w*** fragments upon collisional activation, along with ***c*** and ***y*** fragments that form through the well‐established mechanism for phosphodiester backbone bond cleavage.^4^, ^10^ Importantly, RTD spectra can be recorded on any mass spectrometer that is equipped with an ESI source and a collision cell for CAD.

The spectra from ESI of solutions of RNA **1** (Table 1) without and with [Co^III^(NH_3_)_6_]^3+^ (see Figure S1 in the Supporting Information) illustrate the efficient formation of (M+Co^III^(NH_3_)_6_−*n* H)^(*n*−3)−^ ions, in agreement with previous studies by Kieltyka and Chow.^11^ Isolation and CAD of (M+Co^III^(NH_3_)_6_−9 H)^6−^ ions (measured monoisotopic *m*/*z* 827.289, calculated *m*/*z* 827.289) at 51 eV produced ions formed by loss of NH_3_, 2 NH_3_, 6 NH_3_, and (5 NH_3_+^.^NH_2_; Figure 1). Moreover, ***a***, ***c***, ***d***, ***y***, and ***w*** fragments were observed, both with and without Co^II^ attached (calculated Δm 56.918 Da, which equals 58.932 Da for Co^2+^ minus 2.015 Da for 2H^+^). Fragments with [Co^III^(NH_3_)_6_]^3+^ attached were a minor fraction (ca. 2 %) and of the ***a***, ***c***, and ***y*** type but not the ***d*** and ***w*** type. Of all the fragments from RNA backbone cleavage (excluding internal fragments^12^ and those from cleavage at sites 1 and 14, as ***d***_1_ and ***w***_1_, and ***d***_14_ and ***w***_14_, of RNA **1** have the same mass, and ***y***_1_ is generally uncharged), about 5 % were ***a***, 6 % ***c***, 37 % ***d***, 9 % ***y***, and 42 % ***w***. In addition to ***c*** and ***y*** fragments, CAD can also produce complementary ***a*** and ***w*** fragments (Scheme 1), especially at high energy and when the RNA anions have a high net charge.^12^, ^13^ However, the similarly high abundances of ***d*** and ***w*** fragments from CAD of (M+Co^III^(NH_3_)_6_−*n*)^(*n*−3)−^ ions of RNA **1** at all energies used (Figure 2 A, Figure S2) suggest that all ***d*** and the majority of ***w*** fragments originated from the same dissociation pathway that—in analogy to RNA dissociation into ***d*** and ***w*** fragments by EDD^5^—involves a radical species.

**Figure 1:**
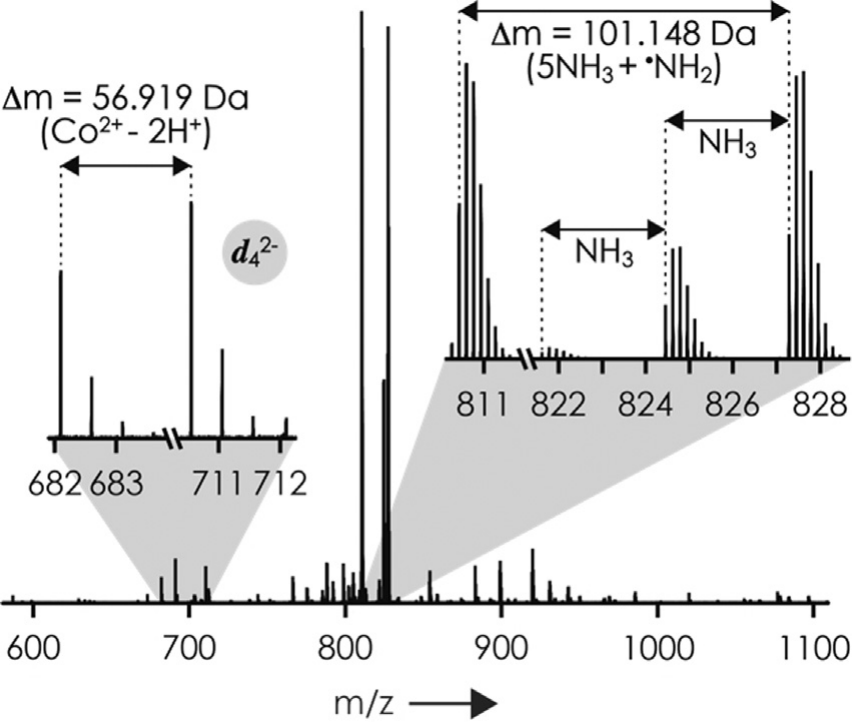
CAD spectrum of (M+Co^III^(NH_3_)_6_−9 H)^6−^ ions of RNA **1** (51 eV laboratory frame collision energy). The insets show signals from loss of NH_3_, 2 NH_3_, and (5 NH_3_+^.^NH_2_), and ***d***_4_^2−^ fragments with and without Co^2+^ attached.

**Figure 2:**
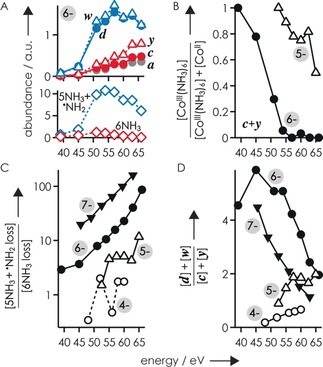
A) Abundances (in arbitrary units) of ***a***, ***c***, ***d***, ***y***, and ***w*** fragments and ions from loss of (5 NH_3_+^.^NH_2_) and 6 NH_3_ by CAD of (M+Co^III^(NH_3_)_6_−9 H)^6−^ ions. B) Fraction of ***c*** and ***y*** fragments with [Co^III^(NH_3_)_6_]^3+^ attached (relative to all ***c*** and ***y*** fragments with Co attached). Branching ratios of C) ions from loss of (5 NH_3_+^.^NH_2_) and 6 NH_3_ and D) ***d***+***w*** and ***c***+***y*** fragments from CAD of (M+Co^III^(NH_3_)_6_−*n* H)^(*n*−3)−^ ions for *n*−3=4–7, versus collision energy.

**Table 1:** RNAs studied.

RNA	Sequence^[a]^	
**1**	GAAGG GCAAC CUUCG	
**2**	GAAGG **DDDD**C CUUCG	**D**: deoxyribospacer
**3**	GAAGG **RRRR**C CUUCG	**R**: ribospacer
**4**	GGUCU GGGCG CAGCG UCAAU GACGC UGACG GUACA GGCC	
**5**	GCGAA CCUGC GGGUU CG	
**6**	GCGAA CCUG**hm^5^C** GGGUU CG	**hm^5^C**: 5‐hydroxymethylcytidine
**7**	GCGAA CCUG**f^5^C** GGGUU CG	**f^5^C**: 5‐formylcytidine

[a] From the 5′‐ to the 3′‐terminus, OH‐terminated.

The ***c*** and ***y*** fragments from CAD of (M+Co^III^(NH_3_)_6_−7 H)^4−^ ions with Co attached all carried [Co^III^(NH_3_)_6_]^3+^, whereas those from CAD of (M+Co^III^(NH_3_)_6_−10 H)^7−^ all carried Co^II^. For the (M+Co^III^(NH_3_)_6_−*n* H)^(*n*−3)−^ ions with *n*−3=5 and 6, the fraction of ***c*** and ***y*** fragments with Co^III^(NH_3_)_6_ attached decreased with increasing energy used for CAD (Figure 2 B), which suggests that phosphodiester backbone bond cleavage into ***c*** and ***y*** fragments (reaction (1), Scheme 2) has lower energy requirements than dissociation of all six NH_3_ molecules. Moreover, the ***c*** and ***y*** fragments with Co^II^ attached must have formed by a mechanism other than phosphodiester backbone bond cleavage and subsequent loss of 6 NH_3_, as the latter cannot account for the change in oxidation state from Co^III^ to Co^II^.

**Scheme 2:**
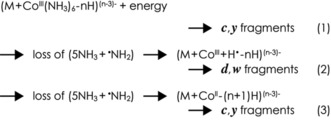
Proposed dissociation reactions in RTD.

With increasing energy and net charge of the (M+Co^III^(NH_3_)_6_−*n* H)^(*n*−3)−^ ions, the number of ions resulting from loss of (5 NH_3_+^.^NH_2_) increased substantially (Figure 2 A), up to about 160‐fold compared to that of the (M+Co^III^−*n* H)^(*n*−3)−^ ions resulting from loss of 6 NH_3_ (Figure 2 C). The ions resulting from loss of (5 NH_3_+^.^NH_2_) could be radical (M+Co^III^+H^.^−*n* H)^(*n*−3)−.^ ions (formed by H^.^ transfer from NH_3_ to the RNA) as well as even‐electron (M+Co^II^−(*n*−1)H)^(*n*−3)−^ ions (formed by electron transfer to Co^III^ and proton transfer to the RNA). As CAD (51 eV) of even‐electron (M+Co^II^−8 H)^6−^ ions from ESI of solutions of RNA **1** with cobalt(II) acetylacetonate did not produce any ***d*** and far fewer ***w*** fragments (***a***: ≈15 %, ***c***: ≈33 %, ***y***: ≈32 %, ***w***: ≈20 %) than CAD of (M+Co^III^(NH_3_)_6_−9 H)^6−^ ions, we conclude that a substantial fraction of the ions formed by loss of (5 NH_3_+^.^NH_2_) are radical (M+Co^III^+H^.^−9 H)^6−.^ ions that can dissociate into ***d*** and ***w*** fragments (reaction (2), Scheme 2). By contrast, the formation of ***c*** and ***y*** fragments that did not carry [Co^III^(NH_3_)_6_]^3+^ likely involved nonradical (M+Co^II^−8 H)^6−^ ions (formed by electron transfer to Co^III^ and proton transfer to the RNA, reaction (3)), as their mass values were consistent with Co^II^ but not Co^III^. Moreover, CAD of (M+Co^III^(NH_3_)_6_−*n* H)^(*n*−3)−^ ions of RNA **2** (which lacks 2′‐OH groups at positions 6–9, Table 1) produced virtually no ***c*** and ***y*** fragments from cleavage at sites 6–9 (Figures S3 and S4), which agrees with the established nonradical mechanism for RNA dissociation into ***c*** and ***y*** fragments that involves the 2′‐OH group.^4^

The steep increase in the number of ions resulting from loss of (5 NH_3_+^.^NH_2_) in CAD of (M+Co^III^(NH_3_)_6_−9 H)^6−^ ions in the energy range 45–57 eV coincided with a steep increase in the number of ***d*** and ***w*** fragments. However, above 57 eV, the number of ***c*** and ***y*** fragments increased, whereas the number of ***d*** and ***w*** fragments decreased (Figure 2 A). These data indicate lower energy requirements for H^.^ transfer and dissociation into ***d*** and ***w*** fragments (reaction (2)) than for separate H^+^ and e^−^ transfer and dissociation into ***c*** and ***y*** fragments (reaction (3)), which is also reflected in the branching ratio between ***d***+***w*** and ***c***+***y*** fragments for *n*−3=6 and 7 (Figure 2 D). For *n*−3=4 and 5, reaction (1) was predominant (Figure 2 B), and the increasing branching ratio between ***d***+***w*** and ***c***+***y*** fragments with increasing energy (Figure 2 D) reflects the competition between reactions (1) and (2). The energy requirements for the reactions in Scheme 2 can thus be ranked as (1)<(2)<(3).

To further test our hypothesis that both radical (M+Co^III^+H^.^−9 H)^6−.^ and even‐electron (M+Co^II^−8 H)^6−^ ions are formed by CAD of (M+Co^III^(NH_3_)_6_−9 H)^6−^ ions of RNA **1**, we used collisional activation in the source region of the instrument, isolated the products resulting from loss of (5 NH_3_+^.^NH_2_) (along with about 7 % (M+Co^III^−9 H)^6−^ ions), and subjected them to CAD (54 eV) in the collision cell. This experiment produced ***a***, ***c***, ***d***, ***y***, and ***w*** fragments, with and without Co^II^ attached, from which we conclude that both radical (M+Co^III^+H^.^−9 H)^6−.^ and even‐electron (M+Co^II^−8 H)^6−^ ions were produced by dissociation of (5 NH_3_+^.^NH_2_) from (M+Co^III^(NH_3_)_6_−9 H)^6−^ ions. The branching ratio between ***d***+***w*** and ***c***+***y*** fragments was about 4.1, which is somewhat lower than that from CAD of (M+Co^III^(NH_3_)_6_−9 H)^6−^ ions at 54 eV without collisional activation in the source region (ca. 5.1) but close to that at 57 eV (ca. 4.2, Figure 2 D), consistent with combined activation in the source and the collision cell.

Our proposed mechanism for the formation of ***d*** and ***w*** fragments by RTD is illustrated in Scheme 3. In the first step, two coordinative bonds between Co^3+^ and two—presumably adjacent—phosphodiester moieties are formed, along with the loss of two NH_3_ molecules. Next, a phosphodiester moiety abstracts H^.^ from a coordinated NH_3_ molecule, while the other three NH_3_ molecules dissociate. The resulting phosphoranyl radical reacts by elimination of both buta‐1,3‐dien‐1‐ol and a nucleobase aldehyde (corresponding to loss of an uncharged nucleoside moiety), along with loss of ^.^NH_2_ and reduction of Co^3+^ to Co^2+^. The latter remains bound by electrostatic interactions to either the ***d*** or the ***w*** fragment after separation of the fragments. For example, about 62 % of the ***d***_4_ (Figure 1) and 39 % of the ***w***_10_ fragments formed by CAD of the (M+Co^III^(NH_3_)_6_−9 H)^6−^ ions of RNA **1** at 51 eV carried Co^2+^ (Figure S5), which adds up to about 100 %. Our proposed mechanism thus provides a rationale for the types of fragments formed (***d*** and ***w***), the unusual loss of (5 NH_3_+^.^NH_2_) from the (M+Co^III^(NH_3_)_6_−*n* H)^(*n*−3)−^ ions, and the reduction of Co^3+^ to Co^2+^ (Figure 1). Experiments with [Ru^III^(NH_3_)_6_]^3+^ instead of [Co^III^(NH_3_)_6_]^3+^, namely CAD of (M+Ru^III^(NH_3_)_6_−*n* H)^(*n*−3)−^ ions of RNA **1**, showed sequential loss of all the NH_3_ ligands and did not produce any ***d*** fragments. Furthermore, the coordinating NH_3_ ligands appear to be critical to H^.^ transfer (Scheme 3), as reactions between dA_6_ anions and cationic *N*,*N′*‐ethylenebis(salicylideneiminato)Co^III^ complexes showed only products corresponding to electron and metal transfer but not H^.^ transfer.^14^

**Scheme 3:**
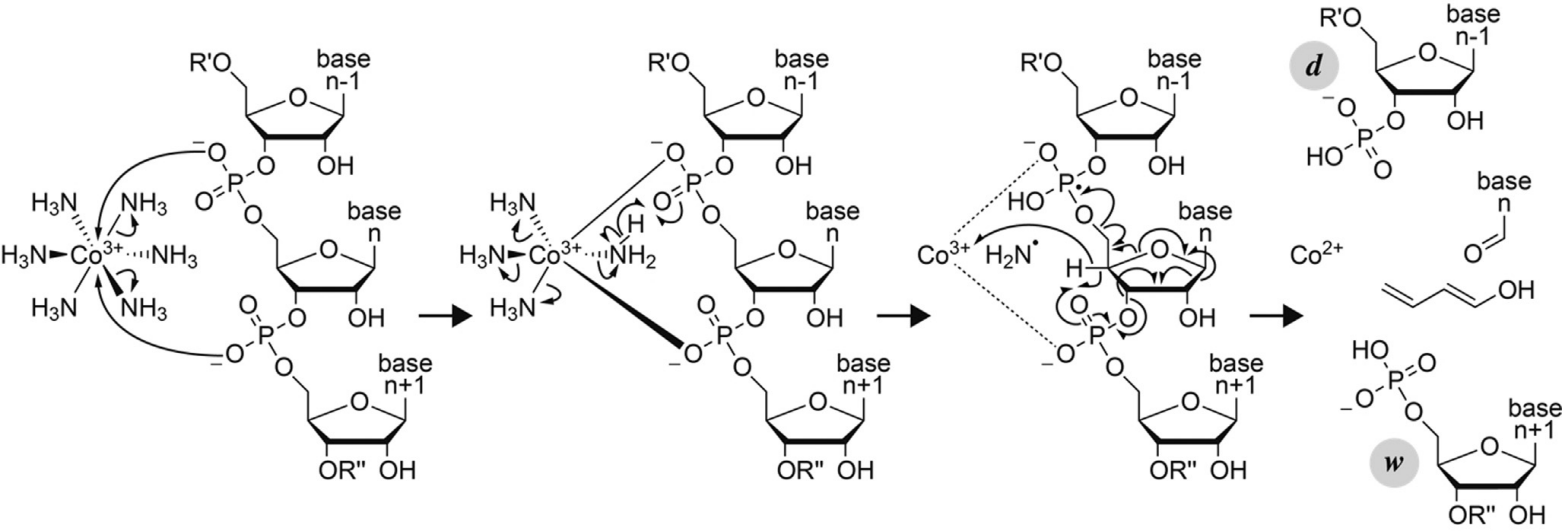
Proposed mechanism for the formation of ***d*** and ***w*** fragments by RTD.

The site‐specific extent of RNA backbone cleavage into ***d*** and ***w*** fragments was not significantly affected by the presence or absence of nucleobases and ribose 2′‐OH groups (Table 1, see also Figures S3 and S4). This observation agrees with our proposed mechanism (Scheme 3), which involves neither the nucleobases nor the ribose 2′‐OH groups. However, the site‐specific extent of dissociation into ***d*** and ***w*** fragments was affected by the net charge of the (M+Co^III^(NH_3_)_6_−*n* H)^(*n*−3)−^ ions (Figures S3 and S4), which we tentatively attribute to different sites of Co(NH_3_)_6_ binding in the (M+Co^III^(NH_3_)_6_−*n* H)^(*n*−3)−^ ions at different net charge. In support of this hypothesis, the occupancy of ***d*** and ***w*** fragments with Co was affected by the net charge (Figure S5).

On extending our new dissociation technique to the larger RNA **4** (39 nt), we found that an increase in the number of [Co^III^(NH_3_)_6_]^3+^ adducts from one to two increased the branching ratio between ***d***+***w*** and ***c***+***y*** fragments from about 0.35 (CAD of (M+Co^III^(NH_3_)_6_−17 H)^14−^ ions at 119 eV) and 0.39 (CAD of (M+Co^III^(NH_3_)_6_−18 H)^15−^ ions at 111 eV) to 0.89 (CAD of (M+2 [Co^III^(NH_3_)_6_]−21 H)^15−^ ions at 105 eV), although all three RTD spectra of RNA **4** provided full sequence coverage (Figure 3). Finally, because RTD into ***d*** and ***w*** fragments involves neither nucleobases nor the 2′‐OH groups (Scheme 3), it should be especially useful for the characterization of modified RNA.

**Figure 3:**
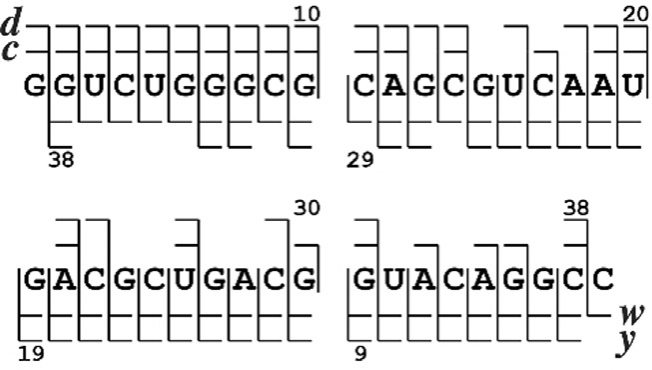
Cleavage map illustrating 100 % sequence coverage from ***c***, ***d***, ***w***, and ***y*** fragments from CAD of (M+2 [Co^III^(NH_3_)_6_]−21 H)^15−^ ions of RNA **4**.

To demonstrate RTD of modified RNA, we studied the 17 nt RNAs **5**, **6**, and **7** with cytidine (**C**), 5‐hydroxymethylcytidine (**hm^5^C**), and 5‐formylcytidine (**f^5^C**) at position 10, respectively (Table 1). CAD of the (M+H)^+^ ions of **C**, 5‐methylcytidine (**m^5^C**), **hm^5^C**, and **f^5^C** indicated that the glycosidic bond of **C** is more stable than those of **m^5^C**, **hm^5^C**, and **f^5^C** by factors of 1.07, 1.25, and 1.74, respectively (Figure S6). CAD of RNAs **5**, **6**, and **7** with **C**, **hm^5^C**, and **f^5^C** at position 10, respectively, showed that losses of **A**, **C**, and **G** nucleobases from (M−*n* H)^*n*−^ ions (base loss from **U** was not observed) were not significantly affected by the presence of **hm^5^C** or **f^5^C**, but that loss of guanine and adenine are favored at lower and higher net negative charge, respectively (Figure S7 A). Moreover, the up to 11‐fold higher base loss from **f^5^C** compared to that from **A**, **C**, and **G** confirmed the low stability of the glycosidic bond of **f^5^C** (Figure S7 B). For RNA **6**, the loss of H_2_O from **hm^5^C** was competitive with nucleobase loss and similar in extent to nucleobase loss from **f^5^C** (Figure S7 C). Notably, the extent of nucleobase and H_2_O loss from fragments from RTD of (M+Co^III^(NH_3_)_6_−10 H)^7−^ ions was generally lower than for CAD of (M−7 H)^7−^ ions of RNAs **5**, **6**, and **7** (Table S1). For example, extensive **f^5^C** nucleobase loss from ***a***_10_ (93 %) and ***c***_10_ (21 %) formed by cleavage of the backbone next to **f^5^C** at position 10 was observed in CAD of RNA **7**, whereas RTD did not produce any ***c***_10_ or ***d***_10_ fragments that showed **f^5^C** nucleobase loss. We conclude that binding of [Co^III^(NH_3_)_6_]^3+^ either increases the stability of the glycosidic bond or lowers the energy required for backbone cleavage below that for nucleobase dissociation.

In conclusion, we report a new dissociation technique, RTD, that allows for de novo sequence characterization of modified RNA without the need for laborious sample preparation or specialized MS instrumentation. As naturally occurring, stable Co is monoisotopic (100 % ^59^Co), the isotope distributions of RNA and RNA fragments with and without Co are highly similar, and existing algorithms can be used for automated data analysis. The unique RTD radical reactions made possible by [Co^III^(NH_3_)_6_]^3+^ considerably expand the repertoire of dissociation techniques for the characterization of RNA by mass spectrometry.

## Experimental Section

Experiments were performed on a 7 T FT‐ICR instrument (Bruker, Austria) equipped with an ESI source, a linear quadrupole for ion isolation, and a collision cell for CAD. RNA was prepared by solid‐phase synthesis, purified by HPLC, desalted,^5^ and electrosprayed from 0.5–2 μm solutions in 1:1 H_2_O/CH_3_OH with ca. 1.25 mm piperidine and 1–2 μm hexamminecobalt(III) chloride (Sigma Aldrich, Austria) at a flow rate of 1.5 μl min^−1^. Data reduction utilized the SNAP2 algorithm (Bruker, Austria).

## Conflict of interest

The authors declare no conflict of interest.

## Supporting information

As a service to our authors and readers, this journal provides supporting information supplied by the authors. Such materials are peer reviewed and may be re‐organized for online delivery, but are not copy‐edited or typeset. Technical support issues arising from supporting information (other than missing files) should be addressed to the authors.

Supplementary
