# Supplementary material for: Radical Transfer Dissociation for De Novo Characterization of Modified Ribonucleic Acids by Mass Spectrometry
Source: Angew Chem Int Ed Engl. 2020 Jan 31;59(11):4309–13. doi: 10.1002/anie.201914275 (PMC7065001; doi:10.1002/anie.201914275)
Supplement: Supplementary file 1 — Supplementary [file ANIE-59-4309-s001.pdf]

Angewandte Chemie
Eine Zeitschrift der Gesellschaft Deutscher Chemiker

Supporting Information

Radical Transfer Dissociation for De Novo Characterization of Modified Ribonucleic Acids by Mass Spectrometry

*Giovanni Calderisi, Heidelinde Glasner, and Kathrin Breuker**

anie_201914275_sm_miscellaneous_information.pdf

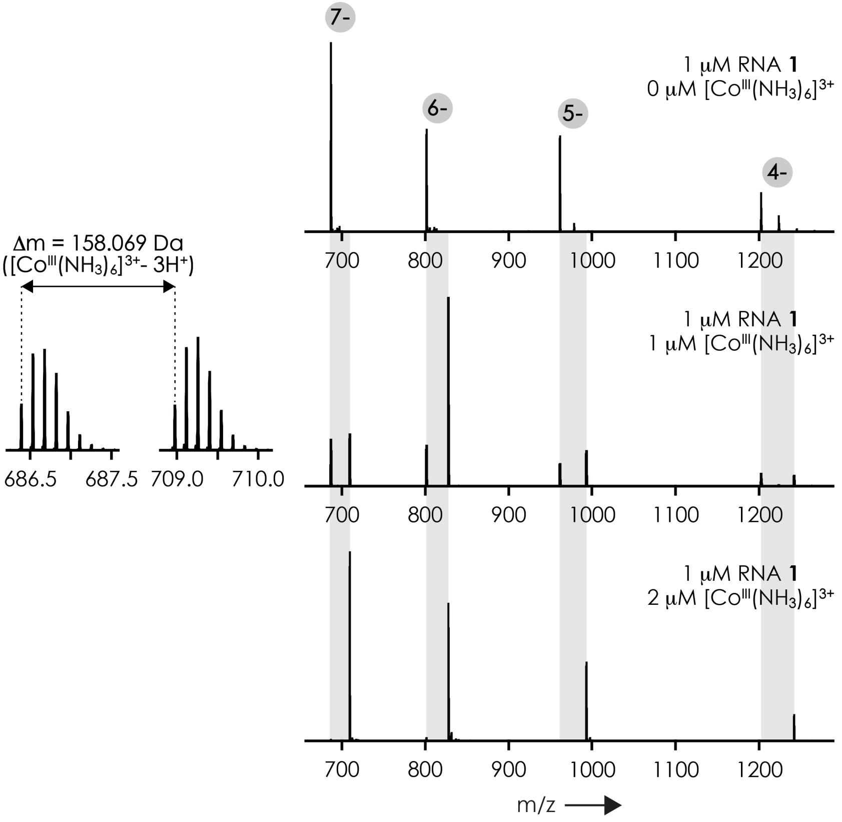

Figure S1. ESI spectra of RNA **1** (1 μM) in 1:1 H_2O/CH_3OH solutions with piperidine (1.2 mM) as ESI additive and 0, 1, or 2 μM $[Co^{III}(NH_3)_6]^{3-}$ as indicated, illustrating efficient formation of $(M + Co^{III}(NH_3)_6 - nH)^{(n-3)-}$ ions.

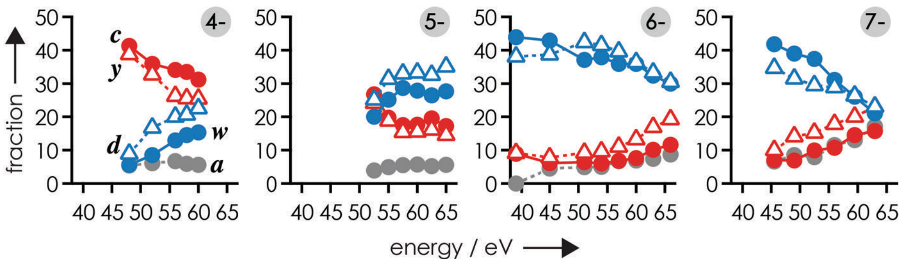

Figure S2. Fractions of ***a***, ***c***, ***d***, ***y***, ***w*** fragments from CAD of $(M + Co^{III}(NH_3)_6 - nH)^{(n-3)-}$ ions of RNA **1** for n-3 = 4-7 (relative to all fragments from RNA backbone cleavage, excluding internal fragments and fragments from cleavage at sites 1 and 14, as d_1 and w_1, and d_{14} and w_{14}, of RNA **1** have the same mass, and y_1 is generally uncharged) versus collision energy.

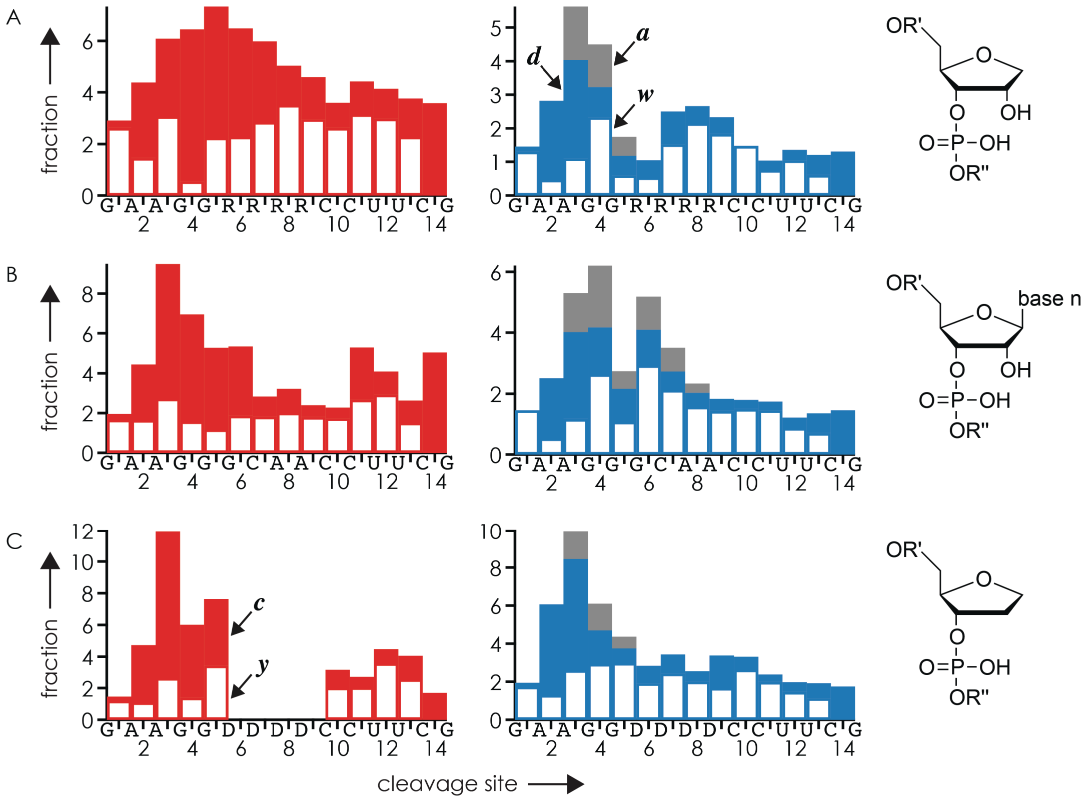

Figure S3. Fractions of ***a***, ***c***, ***d***, ***y***, and ***w*** fragments (relative to all fragments from RNA backbone cleavage, excluding internal fragments) from CAD (56 eV) of $(M + Co^{III}(NH_3)_6 - 7H)^{4-}$ ions of A) RNA **2** (ribose-spacer), B) RNA **1**, and C) RNA **3** (2'-deoxyribose-spacer) versus cleavage site; because d_1 and w_1 (and d_{14} and w_{14}) of RNA **1**, **2**, and **3** have the same mass, the fractions of d_1 and w_1 (and d_{14} and w_{14}) were plotted as 50% of their total.

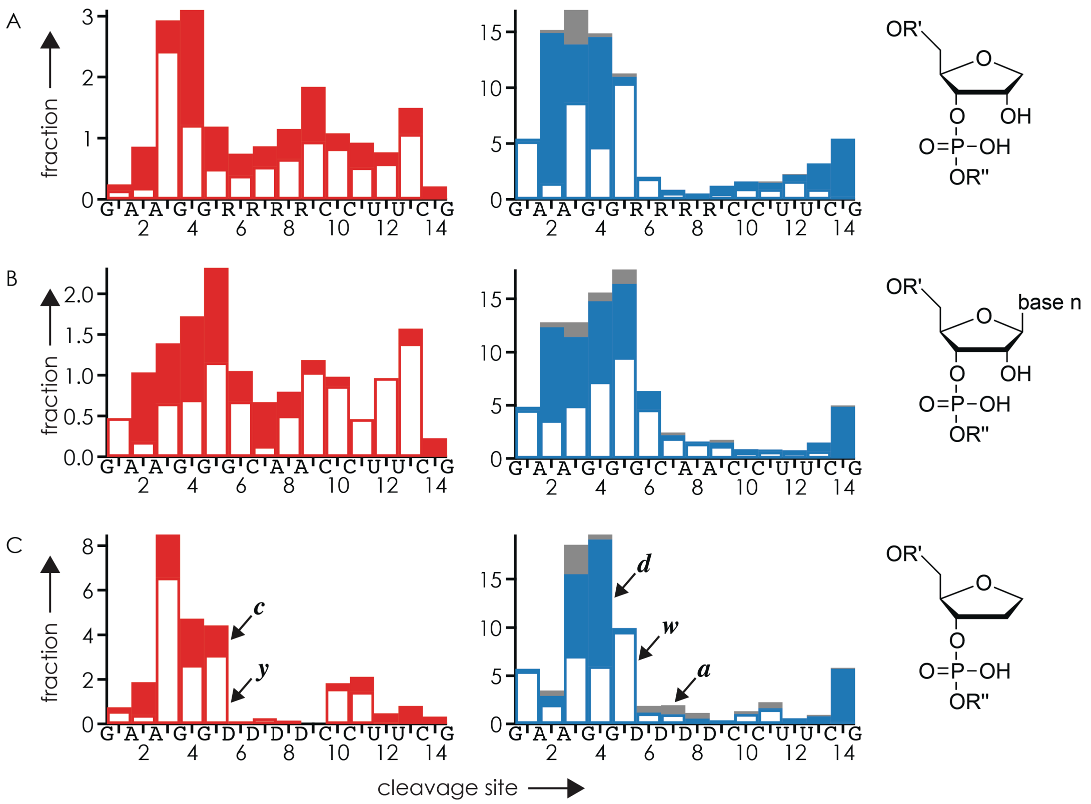

Figure S4. Fractions of ***a***, ***c***, ***d***, ***y***, and ***w*** fragments (relative to all fragments from RNA backbone cleavage, excluding internal fragments) from CAD (54 eV) of $(M + Co^{III}(NH_3)_6 - 9H)^{6-}$ ions of A) RNA **2** (ribose-spacer), B) RNA **1**, and C) RNA **3** (2'-deoxyribose-spacer) versus cleavage site; because d_1 and w_1 (and d_{14} and w_{14}) of RNA **1**, **2**, and **3** have the same mass, the fractions of d_1 and w_1 (and d_{14} and w_{14}) were plotted as 50% of their total.

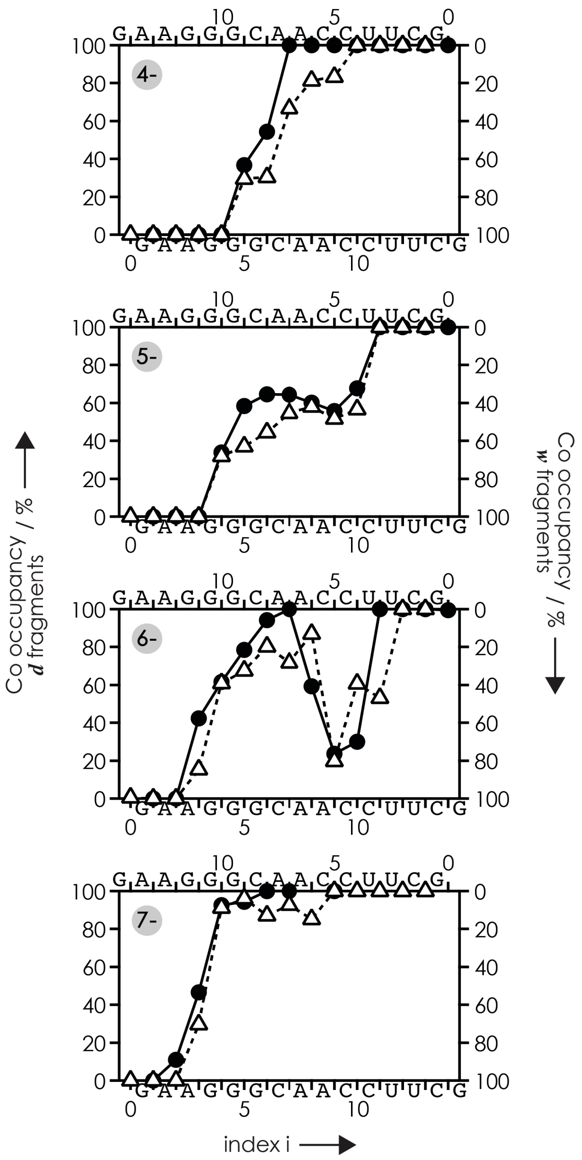

Figure S5. Occupancy of d_i (left axis) and w_i (right axis) fragments from CAD of (M + $Co^{III}(NH_3)_6$ - nH)$^{(n-3)-}$ ions of RNA **1** at 56 eV (n-3 = 4), 55 eV (n-3 = 5), 54 eV (n-3 = 6), and 56 eV (n-3 = 7) versus index i (d_i: bottom axis, w_i: top axis); note that the bottom and top axes are in opposite directions and shifted to account for the fact that d_i and $w_{(15-1-i)}$ are non-complementary fragments (Scheme 1) from the same dissociation event that involves loss of an uncharged nucleoside moiety.

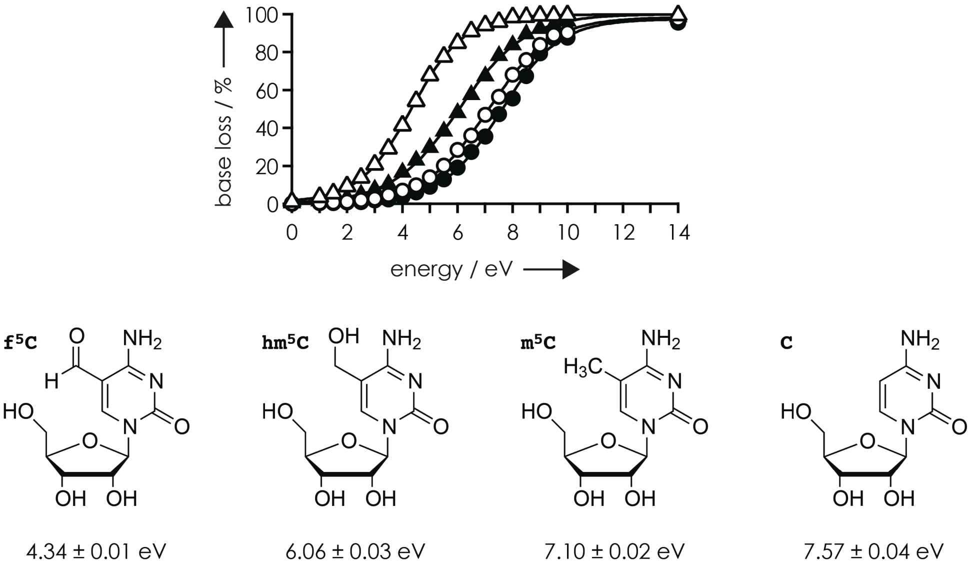

Figure S6. In CAD of $(M + H)^+$ ions of **C** (filled circles), **m⁵C** (open circles), **hm⁵C** (filled triangles), and **f⁵C** (open triangles) nucleosides, percentage of base loss versus laboratory frame energy, with the corresponding E_{50}-values derived from sigmoidal fit functions indicated for each nucleoside.

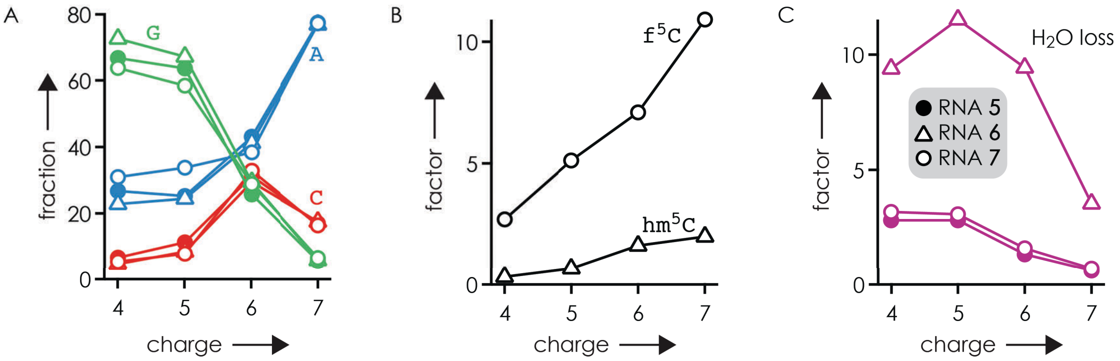

Figure S7. In CAD of $(M\text{-}nH)^{n-}$ ions of RNAs **5** (**C** at position 10, filled circles), **6** (**hm⁵C** at position 10, triangles), and **7** (**f⁵C** at position 10, open circles), A) fraction of nucleobase loss from **A**, **C**, and **G** (normalized to the number of **A** (2), **C** (5 in RNA **5** and 4 in RNAs **5** and **6**), and **G** (7)); B) nucleobase loss from **hm⁵C** and **f⁵C** relative to nucleobase loss from **A**, **C**, and **G**, and C) H_2O loss relative to nucleobase loss from **A**, **C**, and **G**, all versus net charge n.

Table S1. Extent of nucleobase and H_2O loss (in %) from fragments of RNAs **5**, **6**, and **7** in CAD and RTD at 52.5 eV laboratory frame collision energy.

	CAD				RTD			
**RNA**	*a*	*w*	*c*	*y*	*d*	*w*	*c*	*y*
**5** (C10)	64	5	18	5	3	1	4	1
**6** (hm⁵C10)	69	0	11	1	1	1	12	2
**7** (f⁵C10)	75	3	16	7	1	2	10	1
